# Complementary Regulation of BfmRS Two-Component and AbaIR Quorum Sensing Systems to Express Virulence-Associated Genes in *Acinetobacter baumannii*

**DOI:** 10.3390/ijms232113136

**Published:** 2022-10-28

**Authors:** Hyo-Jeong Kim, Na-Yeong Kim, Seo-Yeon Ko, Seong-Yong Park, Man-Hwan Oh, Min-Sang Shin, Yoo-Chul Lee, Je-Chul Lee

**Affiliations:** 1 Department of Microbiology, School of Medicine, Kyungpook National University, Daegu 41944, Korea; 2 Department of Microbiology, College of Science and Technology, Dankook University, Cheonan 16890, Korea

**Keywords:** *Acinetobacter baumannii*, virulence factor, two-component system, quorum sensing system, BfmRS

## Abstract

*Acinetobacter baumannii* expresses various virulence factors to adapt to hostile environments and infect susceptible hosts. This study investigated the regulatory network of the BfmRS two-component and AbaIR quorum sensing (QS) systems in the expression of virulence-associated genes in *A. baumannii* ATCC 17978. The Δ*bfmS* mutant exhibited a significant decrease in surface motility, which presumably resulted from the low expression of *pilT* and *A1S_0112*-*A1S_0119* gene cluster. The Δ*bfmR* mutant displayed a significant reduction in biofilm and pellicle formation due to the low expression of *csu* operon. The deletion of *abaR* did not affect the expression of *bfmR* or *bfmS*. However, the expression of *abaR* and *abaI* was upregulated in the Δ*bfmR* mutant. The Δ*bfmR* mutant also produced more autoinducers than did the wild-type strain, suggesting that BfmR negatively regulates the AbaIR QS system. The Δ*bfmS* mutant exhibited no autoinducer production in the bioassay system. The expression of the *A1S_0112-A1S_0119* gene cluster was downregulated in the Δ*abaR* mutant, whereas the expression of *csu* operon was upregulated in this mutant with a high cell density. In conclusion, for the first time, we demonstrated that the BfmRS-AbaIR QS system axis regulated the expression of virulence-associated genes in *A. baumannii*. This study provides new insights into the complex network system involved in the regulation of virulence-associated genes underlying the pathogenicity of *A. baumannii*.

## 1. Introduction

*Acinetobacter baumannii* causes serious health problems around the world, along with the ‘ESKAPE’ pathogens [1,2]. This Gram-negative pathogen usually infects severely ill patients, causing opportunistic infections in the clinical setting [3]. Its persistence, strengthened by resistance to sanitization and long-term desiccation, and resistance to antimicrobial agents contribute to the successful survival of the organism in a hospital environment [3,4,5]. Moreover, various virulence factors, including outer membrane proteins (OmpA and Omp33-36), lipo-oligosaccharides, capsular polysaccharides, acquisition of metal ions, cell motility, biofilm and pellicle formation, efflux pumps, and outer membrane vesicle formation, play a role in the pathogenesis of *A. baumannii* [6,7]. The expression of virulence-associated genes is controlled by regulatory systems, such as a two-component regulatory system (TCS), quorum sensing (QS) system, histone-like nucleoid-structuring protein, and stringent response regulator DksA, which allow the pathogen to survive in harsh environments and infect susceptible hosts [8,9,10,11].

Bacterial TCS is a key regulatory mechanism that controls the expression of virulence factors, antimicrobial resistance, and adaptation to environmental stimuli [12,13,14]. TCS consists of a sensor kinase that recognizes environmental stimuli and a response regulator that transfers the signals into the cytoplasm [12]. When provoked by environmental or physiological stimuli, the sensor kinase phosphorylates the response regulator. The structural alteration of the response regulator, as a result of phosphorylation, leads to transcriptional regulation of target genes. Five types of TCS, namely, AdeRS, BaeSR, BfmRS, GacSA, and PmrAB, have been well-characterized in *A. baumannii* [8]. BfmR is a response regulator that pairs with BfmS and modulates the expression of virulence-associated genes [8,15]. BfmRS controls gene expression of the K locus, which is composed of genes necessary for capsular exopolysaccharide production [13]. BfmRS also regulates the *csuA/BABCDE* chaperone-usher assembly system, which forms pili involved in the formation of biofilms and pellicles [8,16]. Many studies have been conducted to identify the physiological and pathological roles of BfmRS using mutant strains [13,15,16,17,18,19,20]. BfmR-deficient mutants exhibit a decreased ability in biofilm formation and survival in human ascites and serum [15,17]. Moreover, BfmR increased resistance to certain antimicrobial agents independently of capsular polysaccharides [15]. BfmS-deficient mutants display a decrease in biofilm formation and surface motility, but demonstrate an increase in capsule production [17,19,20]. These diverse phenotypes in the ∆*bfmR* and ∆*bfmS* mutants indicate the multifaceted functions of BfmRS.

The QS system regulates gene expression responding to changes in the surrounding bacterial cell density [9,21]. This system depends on the production and diffusion of signal molecules, autoinducers. In *A. baumannii*, a conventional acyl-homoserine-lactone (AHL)-based QS system has been well-characterized, which consists of a LuxI-type AbaI and LuxR-type AbaR [22,23]. The *abaI* encodes an autoinducer synthase that catalyzes the synthesis of the signal molecule N-hydroxy dodecanoyl-L-homoserine lactone (OHC12-HSL) [24]. The *abaR* encodes a receptor protein that binds autoinducers and acts as a transcriptional regulator. AbaR directly interacts with AHL to form a complex, which binds to a specific DNA sequence, known as a lux box and regulates the expression of target genes [23,24]. The QS system regulates the expression of various virulence factors, including bacterial biofilm formation, pellicle formation, and motility involving *A. baumannii* [23,25]. The ∆*abaR* mutant displays a significant defect in biofilm formation and cell motility, which enable bacteria to persist on abiotic surfaces [22]. Moreover, *abaR* is essential for the expression of virulence factors via *abaI* [22]. Both the TCS and QS systems control many virulence factors simultaneously, but the direct links between TCS and QS systems in the regulation of virulence factors remain unclear in *A. baumannii*. In this study, we investigated the regulatory mechanisms that control the expression of virulence-associated genes in *A. baumannii* via TCS and QS systems. The present study demonstrated that the BfmRS TCS negatively controls the AbaIR QS system, which alters autoinducer production and virulence-associated gene expression in *A. baumannii*.

## 2. Results

### 2.1. Effect of BfmRS on Bacterial Growth and Capsule Production

**Figure 1:**
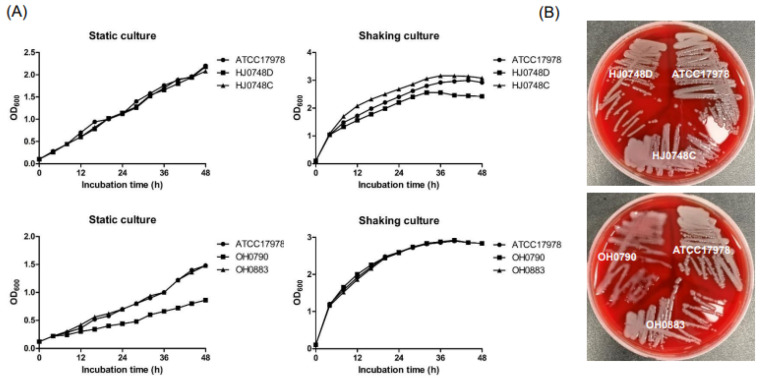
Growth kinetics and capsule production by Δ*bfmR* and Δ*bfmS* mutant strains. (**A**) *A. baumannii* ATCC 17,978 (wild-type), HJ0748D (Δ*bfmR* mutant) HJ0748C (*bfmR*-complemented strain), OH0790 (Δb*fmS* mutant), and OH0883 (*bfmS*-complemented strain) were grown in LB under either shaking or static conditions for 48 h and the OD_600_ was determined at the indicated time points. The data are representative of two experiments with similar results. (**B**) *A. baumannii* strains were grown on blood agar plates for 24 h.

To investigate the role of BfmR in bacterial growth and capsule production, we constructed ∆*bfmR* mutant (HJ0748D) and *bfmR*-complemented (HJ0748C) strains using *A. baumannii* ATCC 17978 (Figure S1A,B). The deletion of *bfmR* (*A1S_0748*) in the HJ0748D strain and its complementation in the HJ0748C strain were confirmed by PCR analysis (Figure S1C). The wild-type (WT) and HJ0748C strains expressed *bfmR*, but little or no expression of *bfmR* was observed in the HJ0478D strain (Figure S1D). The expression of *bfmS* did not differ among the WT, HJ0748D, and HJ0748C strains (Figure S1D), suggesting that the deletion of *bfmR* does not affect the expression of *bfmS*. To determine the effect of BfmR on the growth of *A. baumannii*, the HJ0748D strain was cultured under either shaking or static conditions, and bacterial growth was measured. The WT, HJ0748D, and HJ0748C strains exhibited similar growth patterns under shaking and static conditions; however, under shaking conditions, the HJ0748D strain exhibited a lower optical density at 600 nm (OD_600_) than the WT strain during the post-exponential phase (Figure 1A). Deletion of *bmfS* did not affect the growth rate of *A. baumannii* ATCC 17,978 under shaking conditions, but the growth of the ∆*bfmS* mutant (OH0790) was significantly decreased under static conditions as previously described [19]. To verify the effect of BfmRS on capsular exopolysaccharide production, *A. baumannii* strains were grown on blood agar plates. The OH0790 strain exhibited higher viscosity than the WT strain, as previously described [19], whereas the HJ0748D strain exhibited a phenotype similar to that of the WT strain (Figure 1B). These results suggest that the deletion of *bfmS* affects capsule production and bacterial growth under static conditions, whereas the deletion of *bfmR* has little or no impact on these phenotypes.

### 2.2. Effect of BfmRS on the Surface Motility, Twitching Motility, Biofilm Formation, and Pellicle Formation

**Figure 2:**
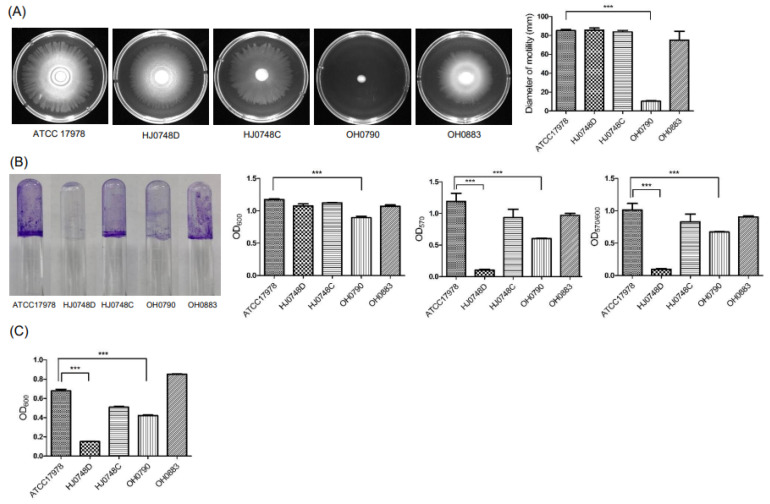
Surface motility, biofilm formation, and pellicle formation of Δb*fmR* and Δb*fmS* mutant strains. (**A**) *A. baumannii* ATCC 17,978 (wild-type), HJ0748D (Δ*bfmR* mutant), HJ0748C (*bfmR*-complemented strain), OH0790 (Δ*bfmS* mutant), and OH0883 (*bfmS*-complemented strain) were inoculated onto the center of motility agar plates and incubated for 12 h at 30 °C. Bacterial migration on the agar plates was measured. The surface motility assay was performed in three independent experiments. (**B**) *A. baumannii* strains were grown in MH medium for 48 h at 30 °C under static conditions. Biofilms were stained with crystal violet. The amount of crystal violet eluted from the biofilms was quantified by measuring the OD_570_. Biofilms were normalized to the total bacterial growth (OD_600_). The data are presented as the mean ± SEM of three independent experiments. (**C**) *A. baumannii* strains were grown in MH medium for 72 h at 30 °C under static conditions. Pellicles were quantitated by measuring the OD_600_. The data are presented as the mean ± SEM of three independent experiments. *** *p* < 0.001 compared to the WT strain.

To assess the effect of BfmRS on the surface motility of *A. baumannii*, the WT, HJ0748D, HJ0748C, OH0790, and OH0883 (*bfmS*-complemented strain) were grown on motility agar plates for 12 h, and the migration distance was measured. The HJ0748D strain moved far from its initial inoculation site, showing a tendency similar to that of the WT strain (Figure 2A). However, the OH0790 strain exhibited little or no surface migration. To determine the effect of BfmRS on the twitching motility of *A. baumannii*, bacterial strains were inoculated in twitching motility plates and were grown for 24 h. The OH0790 strain exhibited a decrease in twitching motility compared with the WT strain. (Figure S2). To determine the effect of BfmRS on biofilm formation by *A. baumannii*, bacterial strains were grown in an Mueller-Hinton (MH) medium for 48 h, and their ability to form biofilms was determined. The biofilm mass (OD_570_/OD_600_) was significantly decreased in both HJ0748D and OH0790 strains (Figure 2B). Growth retardation of the OH0790 strain at OD_600_ was also observed in polystyrene tubes; similar results were obtained when the bacterial were grown in lysogeny broth (LB) under static conditions (Figure 1A). Next, we determined the effect of BfmRS on pellicle formation by *A. baumannii*. The amount of pellicles was significantly decreased in both the HJ0748D and OH0790 strains (Figure 2C), similar to that of biofilm formation. These results suggest that the deletion of *bfmS* results in a significant defect in the surface motility and twitching motility of *A. baumannii*, whereas the deletion of *bfmR* results in a more profound defect in biofilm and pellicle formation than the *bfmS* deletion.

### 2.3. Regulation of Surface-Motility-, Twitching-Motility-, and Biofilm-Associated Genes by BfmRS

**Figure 3:**
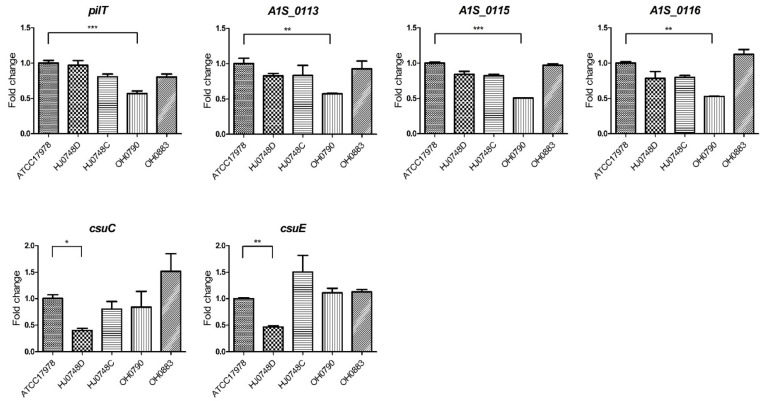
Expression of surface motility- and biofilm-associated genes in Δb*fmR* and Δb*fmS* mutant strains. *A. baumannii* ATCC 17,978 (wild-type), HJ0748D (Δ*bfmR* mutant), HJ0748C (*bfmR*-complemented strain), OH0790 (Δ*bfmS* mutant), and OH0883 (*bfmS*-complemented strain) were grown in MH medium to an OD_600_ of 2.0 and the total RNA was extracted. qPCR was performed to determine the transcription level of genes. The data are the mean ± SEM expression levels of genes in each strain relative to the expression of these genes in ATCC 17978. The data are presented as the mean ± SEM of three independent experiments. * *p* < 0.05, ** *p* < 0.01, *** *p* < 0.001 compared to the WT strain.

To understand the expression and regulation of genes involved in surface motility by BfmRS, the expression of *pilT, A1S_0113*, *A1S_0115*, *A1S_0116*, *csuC*, and *csuE* was analyzed in *A. baumannii* strains. The *pilT* and *A1S_0112*-*A1S_0119* gene clusters are critical for biofilm formation and surface motility [26,27,28]. The *pilT* gene is also responsible for twitching motility of *A. baumannii* [27]. The expression levels of *pilT, A1S_0113*, *A1S_0115*, and *A1S_0116* were not different between the WT and HJ0748D strains, but the expression of these genes was significantly decreased in the OH0790 strain (Figure 3A). The gene expression patterns in the HJ0748D and OH0790 strains were consistent with their surface and twitching motility (Figure 2A and Figure S2). To determine the regulation of biofilm- and pellicle-associated genes by BfmRS, the expression of *csuC* and *csuE* in the *csuA/BABCDE* chaperone-usher pilus system was analyzed. BfmR upregulates the expression of the *csuAB/ABCDE* operon, which is associated with biofilm formation [17,29]. CsuAB pilins are the most abundant substances in pellicles [18]. The expression of *csuC* and *csuE* genes significantly decreased in the HJ0748D strain, but the expression of these genes was not different between the WT and OH0790 strains (Figure 3B); in addition, the expression of *csuC* and *csuE* genes in the HJ0748D strain was consistent with biofilm and pellicle formation (Figure 2B,C). Our results suggest that BfmR controls biofilm and pellicle formation in *A. baumannii* via the *csu* operon, as previously described [16,18], whereas BfmS modulates the expression of *pilT* and *A1S_0112*-*A1S_0119* gene cluster, but not the *csu* operon.

### 2.4. Effect of BfmRS on the Production of Autoinducers

**Figure 4:**
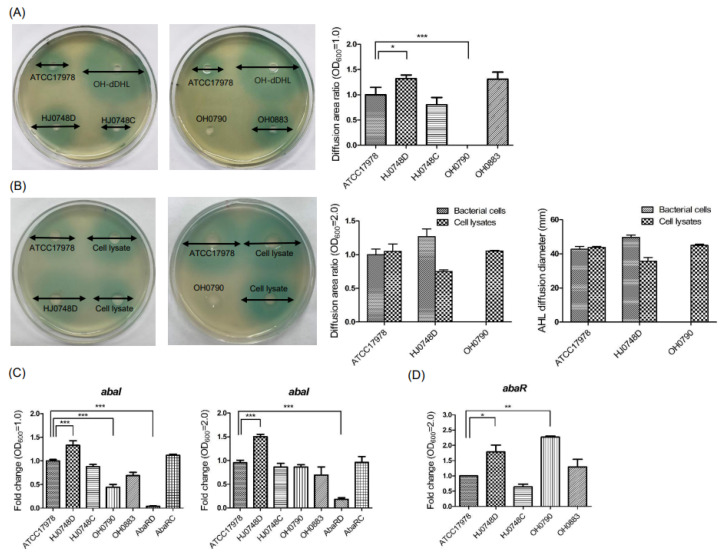
The production of autoinducers and expression of *abaR* and *abaI* in Δ*bfmR* and Δ*bfmS* mutant strains. (**A**) *A. baumannii* ATCC 17,978 (wild-type), HJ0748D (Δ*bfmR* mutant), HJ0748C (*bfmR*-complemented strain), OH0790 (Δ*bfmS* mutant), and OH0883 (*bfmS*-complemented strain) were cultured in LB without salt overnight. Ten microliters of the diluted sample, with fresh LB to an OD_600_ of 1.0, were loaded onto the bioassay agar plates and were incubated for 22 h. Synthetic N-decanoyl-DL-homoserine lactone N-(3-hydroxydodecanoyl)-L-homoserine lactone (OH-dDHL) was used as a positive control. The experiments were performed three times independently. (**B**) *A. baumannii* strains were cultured in LB without salt overnight. Ten microliters of the diluted sample using fresh LB to an OD_600_ of 2.0 and 80 μL cell lysates were inoculated onto the plates overlaid with *A. tumefaciens* NT1 (pDCI41E33) for 22 h and the color zone developed was measured. (**C**) *A. baumannii* strains were grown in LB without salt to an OD_600_ of 1.0 or 2.0 under static conditions. qPCR was performed to analyze the expression of *abaI* and *abaR*. The data are presented as the mean ± SEM of three independent experiments. * *p* < 0.05, ** *p* < 0.01, *** *p* < 0.001 compared to the WT strain.

To investigate the effect of BfmRS on the QS system in *A. baumannii*, autoinducer production was determined using a bioassay. The HJ0748D strain produced more autoinducers than did the WT strain, whereas the OH0790 strain failed to produce autoinducers (Figure 4A). Next, we determined whether OH0790 could produce autoinducers using a high cell density. *A. baumannii* strains and cell lysates at an OD_600_ of 2.0 were inoculated onto the bioassay plates. No autoinducer was produced in the spotted samples of the OH0790 strain (Figure 4B). However, a colored zone was observed in the cell lysates of the OH0790 strain at a high cell density, suggesting that the OH0790 strain could produce autoinducers. To determine whether BfmRS could genetically control autoinducer production, the expression of *abaI* and *abaR* was analyzed in a bacterial growth-dependent manner (OD_600_ 1.0 and 2.0). The expression of *abaI* was significantly upregulated in the HJ0748D strain at OD_600_ 1.0 and 2.0 (Figure 4C). The expression of *abaI* was significantly downregulated in the OH0790 strain when its growth reached an OD_600_ of 1.0, but its *abaI* expression in the same strain was similar to that in the WT counterpart when the bacteria were cultured to an OD_600_ of 2.0 (Figure 4C). The expression of *abaR* was significantly upregulated in the HJ0748D strain (Figure 4D). These results suggest that BfmR negatively regulates autoinducer production in *A. baumannii* through *abaR* and *abaI*.

### 2.5. Effect of AbaR on the Expression of Biofilm- and Surface-Motility-Associated Genes

**Figure 5:**
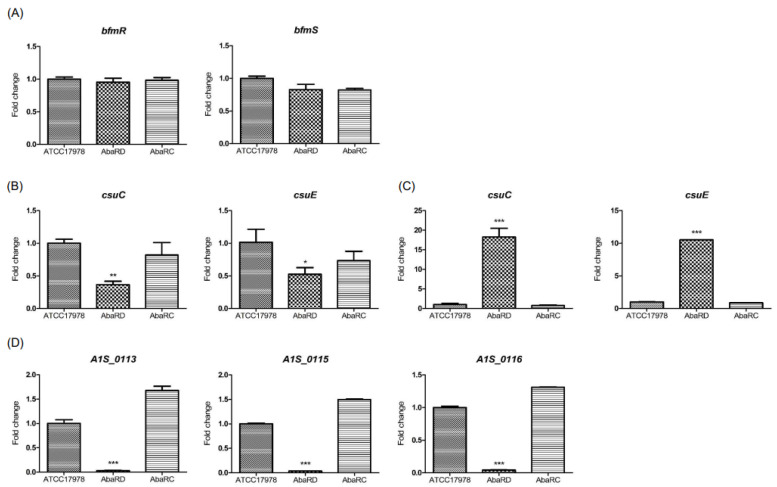
Gene expression in the Δ*abaR* mutant strain. *A. baumannii* ATCC 17,978 (wild-type), AbaRD (Δ*abaR* mutant), and AbaRC (*abaR*-complemented strain) were grown in LB without salt to an OD_600_ of 1.0 or 2.0 under static conditions. qPCR was performed to analyze the expression of genes. (**A**) The expression of *bfmR* and *bfmS* in the Δ*abaR* mutant strain cultured to an OD_600_ of 2.0. (**B**) The expression of *csuC* and *csuE* in the Δ*abaR* mutant strain cultured to an OD_600_ of 1.0. (**C**) The expression of *csuC* and *csuE* in the Δ*abaR* mutant strain cultured to an OD_600_ of 2.0. (**D**) The expression of *A1S_0113*, *A1S_0115*, and *A1S_0116* in the Δ*abaR* mutant strain cultured to an OD_600_ of 2.0. The data are presented as the mean ± SEM of three independent experiments. * *p* < 0.05, ** *p* < 0.01, *** *p* < 0.001 compared to the WT strain.

The quantitative real-time PCR (qPCR) was performed to determine whether AbaR could affect the expression of *bfmR* and *bfmS* in the AbaRD (∆*abaR* mutant) and AbaRC (*abaR*-complemented) strains. The expression of *bfmR* and *bfmS* was not different between the WT and AbaRD strains (Figure 5A). To determine the effect of AbaR on the expression of virulence-associated genes, the expression of *csu* and *A1S_0112-A1S_0119* operons was analyzed. The expression of *csuC* and *csuE* was significantly downregulated in the AbaRD strain cultured to an OD_600_ of 1.0 (Figure 5B), but gene expression was significantly upregulated in the AbaRD strain cultured to an OD_600_ of 2.0. (Figure 5C). The expressions of *A1S_0113*, *A1S_0115*, and *A1S_0116* were significantly downregulated in the mutant strain (Figure 5D). These results suggest that the deletion of *abaR* does not affect the expression of *bfmR* and *bfmS*. Whereas AbaR negatively regulates the expression of the *csu* operon, it positively regulates the expression of *A1S_0112-A1S_0119* operon when a high cell density of *A. baumannii* is used.

## 3. Discussion

**Figure 6:**
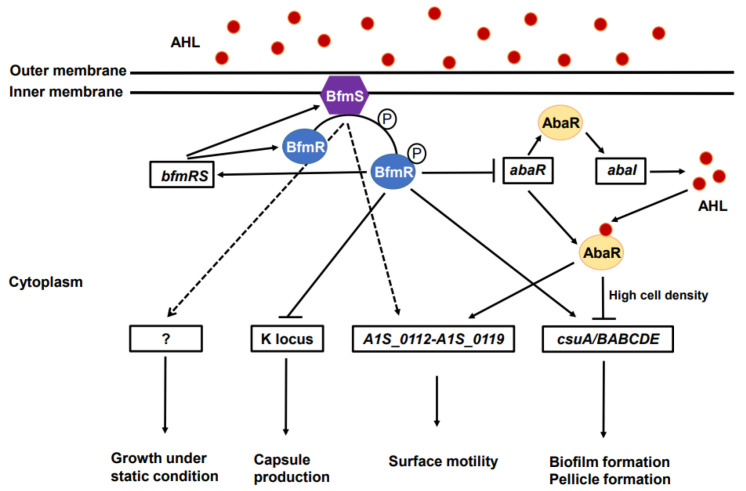
Model of the regulatory networks of BfmRS and AbaIR systems in *A. baumannii*. The solid lines show the interaction between the molecules. The arrowheads and hammerheads indicate activation and repression, respectively. The dotted line means either putative or indirect connection. Question mark means other cognate regulators or factors that are yet to be identified. AHL, acyl-homoserine-lactone.

We investigated the roles of BfmS and BfmR to understand the expression of virulence traits in *A. baumannii*. The present study demonstrated that BfmR controls the biofilm and pellicle formation of *A. baumannii* via the *csu* chaperone-usher pilus system, as previously described [16,18], whereas BfmS controls surface and twitching motility via the *pilT* and *A1S_0112*-*A1S_0119* operon. Furthermore, we investigated the regulatory network of the BfmRS and AbaIR QS systems to express virulence-associated genes in *A. baumannii*. We found that deletion of *bfmR* upregulated the expression of *abaR* and *abaI*, whereas deletion of *abaR* did not affect the expression of *bfmR* or *bfmS*, suggesting that BfmR negatively controls the AbaIR QS system. The deletion of *abaR* resulted in the downregulation of the *A1S_0112*-*A1S_0119* operon, but upregulated the expression of the *csu* operon. The proposed regulatory network of the BfmRS and AbaIR QS systems in the expression of virulence factors is shown in Figure 6.

We investigated the effects of BfmRS on bacterial growth, capsule production, surface motility, biofilm formation, and pellicle formation using the Δ*bfmR* and Δ*bfmS* mutant strains. The Δ*bfmS* mutant exhibited retarded growth under static conditions [19], but deletion of *bfmR* did not affect bacterial growth under both static and shaking conditions. Geisinger et al. [17] also showed that deletion of *bfmS* had no effect on bacterial growth under shaking conditions, but the Δ*bfmRS* mutant showed slightly lower growth rates than the WT strain during the post-exponential phase. Our current observations are consistent with their results regarding the growth of the Δ*bfmR* mutant under shaking conditions. The Δ*bfmRS* mutant downregulated many genes associated with cell wall growth, division, and morphogenesis [17], which presumably resulted in the growth phenotype of Δ*bfmR* mutant under shaking conditions. The *bfmRS* operon regulates K locus genes for the capsular exopolysaccharide production in *A. baumannii* [13,17,19]. The deletion of *bfmS*, disruption of key motifs in the histidine kinase domain of *bfmS*, or mutation of residues required for phosphotransfer in *bfmR* lead to constitutive exopolysaccharide production, resulting in hypermucoid colonies [13]. However, complete deletion of *bfmR* or *bfmRS* did not affect the exopolysaccharide production. These findings suggest that the histidine kinase of BfmS negatively regulates BfmR [13]. In this study, we confirm that complete loss of *bfmS*, but not *bfmR*, produces hypermucoid colonies in *A. baumannii*.

The present study showed that the deletion of *bfmS*, but not *bfmR*, exhibited a dramatic reduction in the surface motility of *A. baumannii*. In contrast to surface motility, deletion of *bfmR* exhibited a more profound effect on biofilm and pellicle formation than *bfmS*. Tomaras et al. [16] reported a slight defect in biofilm formation in the Δ*bfmS* mutant. In our previous study; however, the deletion of *bfmS* did not affect biofilm formation of *A. baumannii* ATCC 17,978 [19]. The discrepancy between the two studies, in biofilm formation by the same Δ*bfmS* mutant (OH0790), may be due to the different growth rates in LB and MH media. The Δ*bfmS* mutant exhibited a higher OD_600_ in MH broth than in LB medium; therefore, biofilm cells relative to total bacterial cells (OD_570/600_) differed between the two studies. BfmS was identified to play a key role in the surface motility of clinical *A. baumannii* M2 strain using transposon mutagenesis [30]. The mutant strain, containing the transposon in *bfmS*, exhibited >80% decrease in the surface motility of *A. baumannii* M2. Moreover, *A1S_0113* (a member of the acyl-CoA dehydrogenase family) and *A1S_0115* (a homolog of non-ribosomal peptide synthetase) in the *A1S_0112-A1S_0119* operon were also responsible for biofilm formation and surface motility [28,30]. Transcriptome analysis showed that all genes in the *A1S_0112-A1S_0119* operon, except *A1S_0119*, were significantly upregulated in biofilm cells relative to exponential or stationary phase cells [24]. In the present study, we demonstrated that the deletion of *bfmR* significantly decreased the expression of *csuC* and *csuE*, as previously described [26,29,30], but did not affect the expression of *pilT, A1S_0113*, *A1S_0115*, and *A1S_0116* (RND superfamily transporter). Deletion of *bfmS* significantly decreased the expression of *pilT* and three genes in the *A1S_0112*-*A1S_0119* operon, but did not affect the expression of the *csu* operon. These results indicate that BfmS may affect the expression of *pilT* and *A1S_0112*-*A1S_0119* operon. BfmR and BfmS regulate the *csu* operon differently. Phosphorylation of BfmR by the activation of BfmS stimulates the dimerization of BfmR, which binds to the DNA sequences of target genes, whereas unphosphorylated BfmR binds to its promoter with a high affinity [31,32]. Further studies are required to characterize the regulatory mechanisms of virulence-associated genes, such as *csu* operon, by BfmR, BfmS, or both.

Both the BfmRS TCS and AbaIR QS systems control several virulence traits such as biofilm formation, pellicle formation, and surface motility in *A. baumannii* coordinately. It has been reported that, in *Pseudomonas aeruginosa*, BfmR binds to and represses the promoter of *rhlR* encoding the transcriptional regulator RhlR in the RhlIR QS system [33]. The RqpSR TCS modulates the QS system and pathogenesis in *Burkholderia cenocepacia* [34]. In the present study, the expression of QS-regulated genes such as *A1S_0112*-*A1S_0119* operon was downregulated in the Δ*bfmS* mutant. Based on these observations, the BfmRS TCS may regulate the AbaIR QS system in *A. baumannii*. To determine the direct links between the BfmRS and AbaIR QS systems, autoinducer production and expression of *abaR* and *abaI* were analyzed in Δ*bfmR* and Δ*bfmS* mutant strains. In agreement with the results obtained for *P. aeruginosa* [33], the Δ*bfmR* mutant produced more autoinducers than the WT strain. The regulation of *abaR* and *abaI* was consistent with the autoinducer production in the Δ*bfmR* mutant. Interestingly, autoinducer production was not observed in the Δ*bfmS* mutant using the bioassay system. Because autoinducer production is highly dependent on cell density, autoinducer production and the expression of *abaR* and *abaI* were analyzed in the Δ*bfmS* mutant using a high cell density (OD_600_ of 2.0). Although autoinducer production was not observed in bioassay agar plates inoculated with the Δ*bfmS* mutant, cell lysates of the mutant displayed autoinducer production. No detection of autoinducers in the Δ*bfmS* mutant strain using the bioassay system may be due to the low growth rates of this mutant under static culture conditions, inhibition of autoinducer diffusion by hyperproduction of capsule, or any defect in the diffusion of autoinducers.

The *aba* QS system is necessary to express virulence-associated genes in *A. baumannii* [23,25,35]. The deletion of *abaR* resulted in no expression of *abaI*, which in turn resulted in a significant reduction in biofilm formation, pellicle formation, and surface motility in *A. baumannii* [22,36]. To understand the regulatory mechanisms resulting in several virulence trait defects in the Δ*abaR* mutant, the expression of *csu* and *A1S_0112-A1S_0119* operons was analyzed. The expression of the three tested genes in the *A1S_0112-A1S_0119* operon was downregulated in the Δ*abaR* mutant. Surprisingly, the expression of *csuC* and *csuE* was upregulated in the Δ*abaR* mutant employing high cell density (OD_600_ of 2.0), but downregulated in that employing low cell density (OD_600_ of 1.0). The formation of biofilms and pellicles was significantly decreased in both Δ*abaR* mutant [22] and Δ*bfmR* mutants. Sun et al. [36] reported downregulation of the *csuA/BABCDE* genes in Δ*abaR*, Δ*abaI*, and Δ*abaIR* mutants using transcriptome analysis. The authors also showed that there was no difference in the expression of *bfmR* and *bfmS* between the WT and three mutants of the AbaIR QS system. However, they did not describe the bacterial culture conditions in the transcriptome analysis and qPCR experiments. Luo et al. [37] demonstrated the upregulation of *csuA/BABCDE*, *bfmR*, and *bfmS* in *A. baumannii* ATCC 19,606 strain grown in LB with 100 μM N-hexanoyl-L-homoserine lactone under shaking conditions for 12 h at 37 °C. In the present study, to determine the expression of virulence-associated genes in the Δ*abaR* mutant, *A. baumannii* strains were grown in LB under static conditions until the stationary phase (OD_600_ of 2.0) at 30 °C to achieve the stability of autoinducers [38]. Differences in experimental conditions may result in different gene expression patterns between studies. Since the *csuA/BABCDE* chaperone-usher pilus system contributes to biofilm formation in *A. baumannii* during early growth stages [29], upregulation of *csuC* and *csuE* in the Δ*abaR* mutant at high cell density cannot play a role in the production of biofilm mass. Intriguingly, the present study demonstrated that the BfmRS, AbaIR QS system, and/or BfmRS-AbaIR QS system controlled the *csuA/BABCDE* chaperone-usher pilus system. Many virulence-associated genes have been identified in *A. baumannii* using transcriptome analysis and transposon mutagenesis; therefore, further studies are needed to identify the virulence-associated genes regulated by the BfmRS-AbaIR QS system axis.

In the present study, we explored possible links between the BfmRS TCS and AbaIR QS systems in the regulation of virulence-associated genes in *A. baumannii*. Virulence traits such as biofilm formation, pellicle formation, and surface motility are sophisticatedly controlled by the BfmRS, AbaIR QS system, and/or BfmRS-AbaIR QS system axis. Although the regulatory systems that control the expression of virulence factors such as BfmR and AbaR are considered potential targets for the development of anti-virulence agents, this study provides insights into the complex network system underlying the pathogenicity of *A. baumannii*.

## 4. Materials and Methods

### 4.1. Bacteria, Plasmids, Culture Media, and Growth Conditions

The bacterial strains and plasmids used in this study are listed in Table S1 [39]. *Escherichia coli* and *A. baumannii* strains were grown in LB at 37 °C unless specified otherwise. The MH (BD Difco, Franklin Lakes, NJ, USA) and *Agrobacterium* minimal media were used to culture *A. baumannii* and *Agrobacterium tumefaciens*, respectively. *A. baumannii* strains were grown on blood agar plates containing 5% sheep blood to analyze capsule production. Agar powder (Junsei Chemical Co., Chuo-ku, Japan), Bacto agar (BD Difco), and Eiken agar (Eiken Chemical Co., Tokyo, Japan) were used. Kanamycin (50 μg/mL), chloramphenicol (20 μg/mL), or carbnicillin (100 μg/mL) were added to the culture medium to select for the mutant or complementary colonies.

### 4.2. Construction of the ΔbfmR Mutant and bfmR-Complemented Strains

The ∆*A1S_0748* (*bfmR*) mutant (HJ0748D) of *A. baumannii* ATCC 17,978 was constructed using a markerless gene deletion method as previously described [40]. The complementation of *bfmR* in the HJ0748D strain was conducted using overlap extension PCR and the *bfmR*-complemented strain (HJ0748C) was constructed (Table S1). PCR primers used in this study are listed in Table S2. The construction of mutant and complemented strains is presented in Figure S1 and Supplementary Materials and methods.

### 4.3. Bacterial Growth Studies

Bacterial strains of *A. baumannii* ATCC 17978, HJ0748D, HJ0748C, OH0790 [19], and OH0883 [19] were used in the present study. Each bacterial strain was grown overnight in LB medium at 37 °C. Bacterial cultures were adjusted in fresh LB to an OD_600_ of 1.0 using a spectrophotometer (Biochrom, Cambridge, UK). The samples were diluted at 1:20 in fresh LB medium and incubated, under shaking or static conditions, at 37 °C for 48 h. The OD_600_ was measured at the indicated time points using a spectrophotometer.

### 4.4. RNA Isolation and Gene Expression Assay Using qPCR

*A. baumannii* strains were grown in MH broth to an OD_600_ of 1.0 or 2.0, at 30 °C or 37 °C under static conditions. Total RNA from each bacterial strain was isolated using an RNeasy Mini Kit (Qiagen, Valencia, CA, USA). The mRNA samples (1.5 μg) were used as templates for the synthesis of complementary DNA (cDNA) using random hexamer primers and the TOPscript™ cDNA synthesis kit (Enzynomics, Daejeon, Korea). Synthesized cDNA was used as a template for qPCR. Target genes were amplified using TOPreal™ qPCR 2xPreMIX (SYBR Green with high ROX) (Enzynomics) using specific primers (Table S3). Gene amplification was performed using a StepOnePlus Real-Time PCR System (Applied Biosystems, Foster City, CA, USA). Gene expression was calculated by normalizing with the 16S rRNA expression in each sample, and the ΔΔCt method was used to determine the fold changes of target genes. The assays were performed in three independent experiments.

### 4.5. Surface Motility Assay

The surface motility was examined on motility agar plates containing 1% (*w*/*v*) tryptone, 0.5% (*w*/*v*) yeast extract, and 0.35% (*w*/*v*) Eiken agar powder. *A. baumannii* strains were grown in LB overnight with shaking at 37 °C. Bacterial cells were diluted with fresh LB to an OD_600_ of 1.0. Bacterial suspension (1 μL) was inoculated onto the center of motility agar plates. The plates were incubated at 30 °C for 12 h, and bacterial migration on the agar plates was photographed using a digital imaging system (WSE-5200, Printgraph 2M, Atto Co., Tokyo, Japan). The surface motility assay was performed in three independent experiments.

### 4.6. Biofilm and Pellicle Formation Assays

*A. baumannii* strains were grown in MH broth overnight at 30 °C. Each bacterial culture was diluted at 1:200 in fresh MH broth. Two milliliters of the diluted culture were inoculated into a polystyrene tube (12 × 75 mm) containing MH broth and incubated for 48 h at 30 °C without shaking. After removing the planktonic cells, the tubes were carefully washed with 2 mL of sterile distilled water. Biofilms formed on the tube walls were stained with 0.1% (*w*/*v*) crystal violet (Junsei Chemical Co., Chuo-ku, Japan) solution for 15 min. The tube was washed twice with 2 mL of sterile distilled water and dried at room temperature. Crystal violet was eluted with 2 mL of 30% acetic acid (Duksan, Gyeonggi-do, Korea) and the biofilms at OD_570_ were measured. The biofilms were normalized to the total bacterial growth at OD_600_. The pellicle formation assay was performed as previously described [22] with minor modifications. *A. baumannii* strains were grown in MH broth overnight at 30 °C. Each bacterial culture was diluted at 1:200 using the fresh medium, and bacterial suspension (10 μL) was inoculated into a polypropylene conical tube (30 × 115 mm) containing MH broth. The tubes were then incubated for 72 h at 30 °C under static conditions. Methanol was added to separate the pellicles from the tubes. The pellicle biomass was quantified by measuring the OD_600_ of the bacteria suspended in 1 mL of phosphate-buffered saline (Welgene, Gyeongsangbuk-do, Korea). Biofilm and pellicle formation assays were performed in three independent experiments

### 4.7. Bioassay for the Detection of Autoinducers

*A. baumannii* strains were grown overnight in LB at 37 °C and diluted using fresh LB to an OD_600_ of 1.0 or 2.0. Ten microliters of the diluted bacterial sample were loaded onto a plate overlaid with *A. tumefaciens* NT1 (pDCI41E33). Synthetic N-decanoyl-DL-homoserine lactone N-(3-hydroxydodecanoyl)-L-homoserine lactone (OH-dDHL) (40 μM) (Sigma-Aldrich, St. Louis, MO, USA) was used as a control. The plates were then incubated for 22 h at 30 °C. Autoinducer production was quantified based on the measurement of the colored areas surrounding the bacteria. To detect autoinducer production in the bacterial lysates, *A. baumannii* strains were grown in 5 mL of LB to an OD_600_ of 2.0. After centrifugation of bacterial cultures, Bacteria were lysed by sonication (Branson Sonifier 450; Danbury, CT, USA). After removing bacteria and cell debris, the supernatants (80 μL) were inoculated onto an agar plate overlaid with *A. tumefaciens* NT1 (pDCI41E33). Three independent bioassays were performed. The preparation of media and bioassays employed for the detection of autoinducers are presented in the Supplementary Materials and methods.

### 4.8. Statistical Analysis

Data were analyzed using the GraphPad Prism software (version 5.0; San Diego, CA, USA). The averages and standard errors of the means (SEM) were calculated. Data from different experimental groups were analyzed using one-way ANOVA with Dunnett’s post hoc analysis or Student’s *t*-test. *p* values of < 0.05 were considered statistically significant.

## Data Availability

The authors confirm that the data supporting the findings of this study are available within the article and its Supplementary Materials.

## Supplementary Materials

The following supporting information can be downloaded at: https://www.mdpi.com/article/10.3390/ijms232113136/s1.
