# Supplementary material for: Complementary Regulation of BfmRS Two-Component and AbaIR Quorum Sensing Systems to Express Virulence-Associated Genes in Acinetobacter baumannii
Source: Int J Mol Sci. 2022 Oct 28;23(21):13136. doi: 10.3390/ijms232113136 (PMC9657202; doi:10.3390/ijms232113136)
Supplement: Supplementary file 1 [file ijms-23-13136-s001.zip › ijms-1949584-supplementary.pdf]

Supplementary Figures

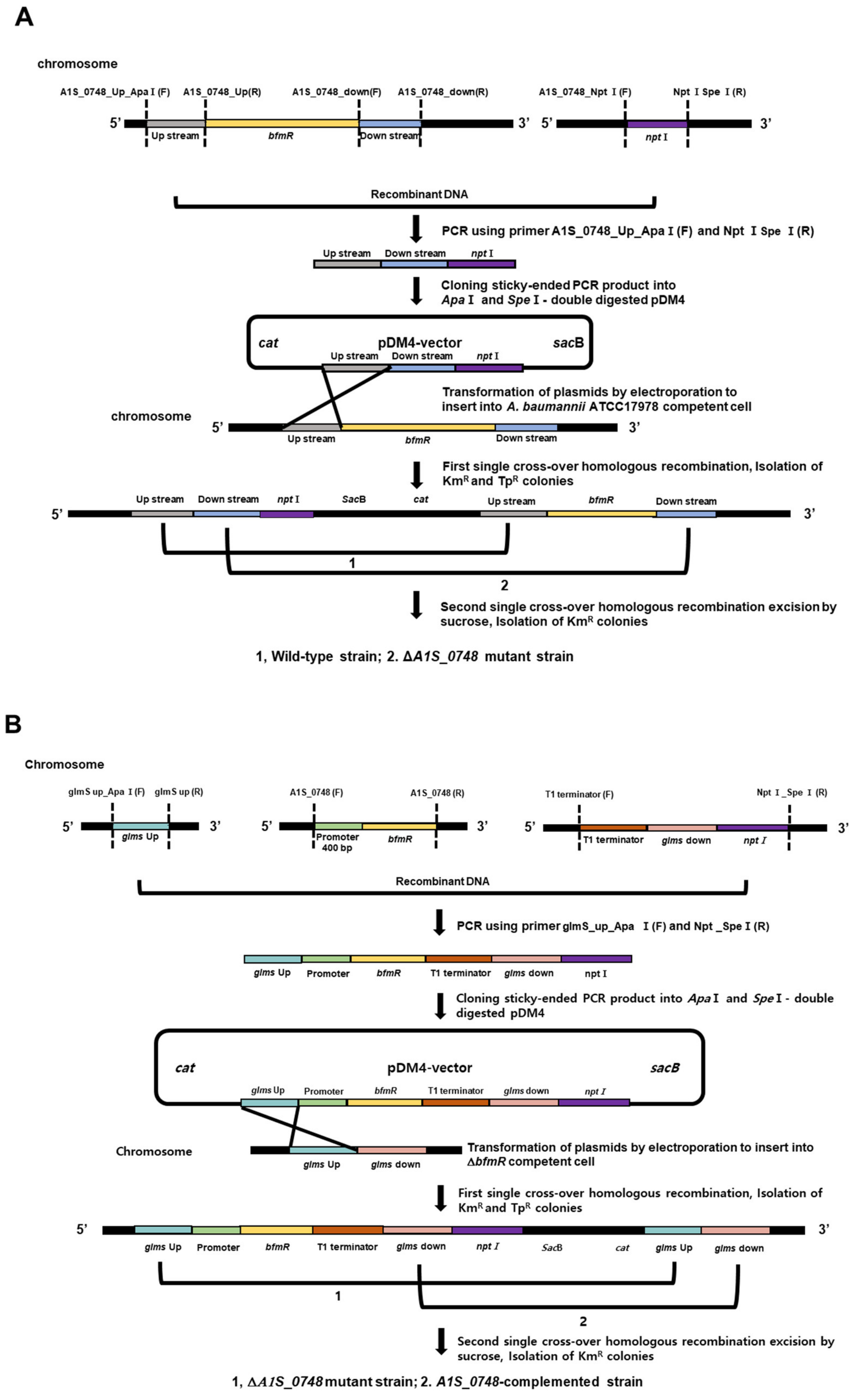

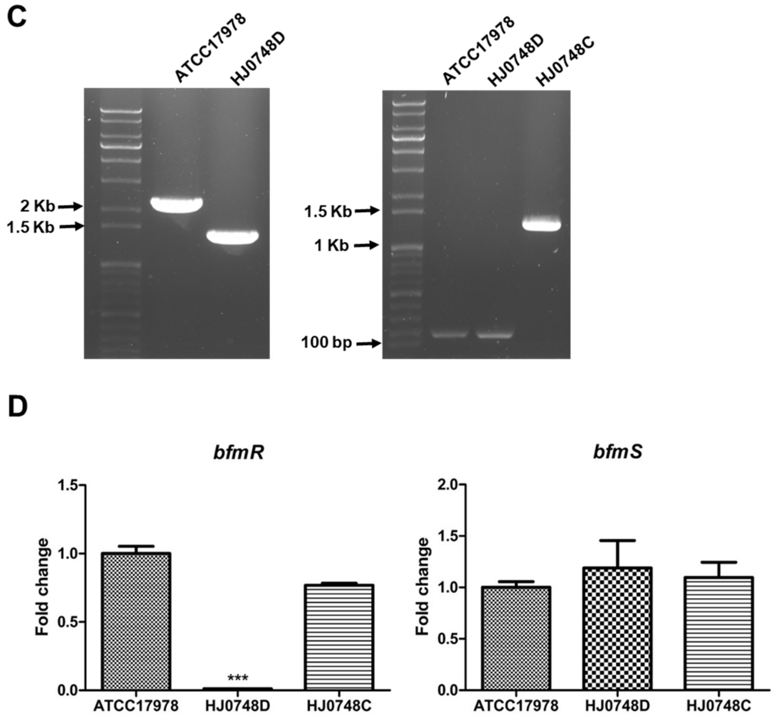

Figure S1. Construction of Δ*bfmR* mutant and its *bfmR*-complemented strains. (A) Overall scheme demonstrating the markerless gene deletion method for the construction of Δ*bfmR* mutant (HJ0748D) in *A. baumannii* ATCC 17978. (B) Overall scheme demonstrating the overlap extension PCR method for the construction of *bfmR*-complemented strain (HJ0748C) in HJ0748D strain. (C) The deletion of *bfmR* in the HJ0748D strain was confirmed by PCR using the primers A1S_0748_Spe I UF and A1S_0748_ DR (Supplementary Table S2). The expected amplicon sizes of 2,140 bp and 1,423 bp were identified in the WT and HJ0748D strains, respectively, by gel electrophoresis. The complementation of *bfmR* in the HJ0748C was confirmed by PCR using the primers glms up_seq_F and glms down_seq_R (Supplementary Table S2). The insertion of *bfmR* in the HJ0748C strain was identified using the amplicon size of 1,227 bp. (D) The expression of *bfmR* and *bfmS* was determined in the WT, HJ0748D, and HJ0748C strains using qPCR employing specific primers (Supplementary Table S3). The data are presented as the mean ± SEM of three independent experiments. *** $p < 0.001$ compared to the WT strain.

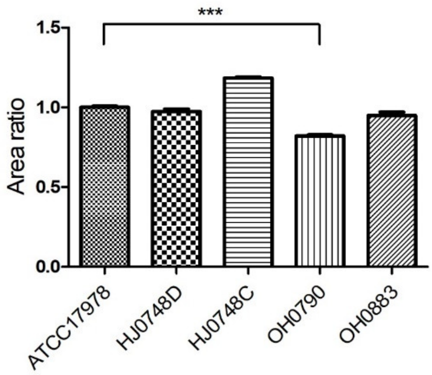

Figure S2. Twitching motility of Δb*fmR* and Δb*fmS* mutant strains. *A. baumannii* ATCC 17978, HJ0748D (Δb*fmR* mutant), HJ0748C (*bfmR*-complemented strain), OH0790 (Δb*fmS* mutant), and OH0883 (*bfmS*-complemented strain) were inoculated by stabbing at the agarose and plate interface and plates were incubated for 24 h. The agarose was removed from plates and bacteria adhered to bottom of plates were stained by crystal violet. The movement area was measured using ImageJ program and the ratio relative to the wild-type strain was calculated. The experiments were performed three times independently. *** $p < 0.001$ compared to the WT strain.

Supplementary Tables

Table S1. Bacterial strains and plasmids used in this study.

Bacteria/plasmids	Relevant characteristics*	Reference or source
A. baumannii		
ATCC 17978	Wild-type strain	ATCC
HJ0748D	Δ*bfmR* of *A. baumannii* ATCC 17978	This study
HJ0748C	*bfmR* with T1 terminator in HJ0748D	This study
OH0790	Δ*bfmS* of *A. baumannii* ATCC 17978	[1]
OH0883	*bfmS* rescued in OH0790	[1]
AbaRD	Δ*abaR* of *A. baumannii* ATCC 17978	[2]
AbaRC	*abaR* rescued in Δ*abaR* with pOMH17	[2]
E. coli		
SY327 λ pir	*supE44 ΔlacU169* (ϕ80 *lac*ZΔM15) *hsdR17 recA1 endA1 gyrA96 thi-1 relA1 λ* pir (phage lysogen); plasmid replication	Laboratory collection
Plasmids		
pOH4	pHKD01 with *ompA* coding region of *A. baumannii* ATCC 17978 under control of its native promoter with *nptI*; Km^r	[3]
pDM4	Suicide vector, *ori*R6K; Cm^R, *sacB*	GenBank accession no. KC795686
pHJ0748D	pDM4 with Δ*bfmR::npt*I; Cm^R, Km^R	This study
pHJ0748C	pDM4 with *glms*, *bfmR*, and *npt*I	This study

* Abbreviations. Km^R, kanamycin-resistant; Cm^R, chloramphenicol-resistant.

Table S2. Oligonucleotides used in this study.

Primers	Sequence (5′→3′)*
Deletion of *bfmR* in *A. baumannii* ATCC 17978	
A1S_0748_Spe I UF	GGACTAGTCAGTACCATTTCCGTCAGTTG
A1S_0748_ UR	GGAAGTTTAATCAGATTTTACATATCATTGCCCCTATAAATCTCATTAC
A1S_0748_ DF	GGGGCAATGATATGTAAAATCTGATTAAACTTCCTATAAGGTTGG
A1S_0748_ DR	GCAGACGCGCAGGATGATTCGG
Npt I_A1S_0748 F	CATCCTGCGCGTCTGCCTCGTGAAGAAG
Npt I_Apa I R	GTTGGGCCCGATCCGTCGACCTGCAGG
Single-copy complementation of *bfmR* in the Δ*bfmR* mutant of *A. baumannii*	
glms_up_Apa I F	GTTGGGCCCTCCTTTGAGCAATTGACTTG
glms_up_A1S_0748 R	GTAAAAAATCCCGGCCTTAATAATGATCTTTTTTGAATTACTCTAC
A1S_0748_glms_up F	CAAAAAAGATCATTATTAAGGCCGGGATTTTTTACGGTAGATCAATC
A1S_0748_T1 R	CGTTTTATTTGATGCCTTACAATCCATTGGTTTCTTTAACAAAC
T1_A1S_0748 F	CCAATGGATTGTAAGGCATCAAATAAAACGAAAGGC
Npt I_Spe I R	GTTACTAGTGATCCGTCGACCTGCAGG
glms_up_seq F	TGGCGAAGTCAGTAACTGTAGA
glms_down_seq R	GGAAAGCTTCTGTGTAGCGATATG

*Underlined sequences indicate regions that are not complementary to the templates.

Table S3. Primers used in qPCR.

Primers	Sequence (5′→3′)	Target genes
16S rRNA F	GCACAAGCGGTGGAGCAT	16S rRNA
16S rRNA R	CGAAGGCACCAATCCATCTC	
bfmR F	GTTTAACCGTTTGTCGTG	*bfmR*
bfmR R	GTGGTTGAACTGGTTTCG	
bfmS F	TTGAACTTATTCCACCGCCTTT	*bfmS*
bfmS R	GCCCGTAATCCGAACTTTGTT	
abaI F	CTACTACCCACCACACAACCCTATTT	*abaI*
abaI R	GGTGAGCAGGGAATAGGCATT	
abaR F	GGTCGAGTCAATCTGCAAAGA	*abaR*
abaR R	CTGAGCCCAACCGACATTTA	
csuC F	AAAGCAGGCGAGAAGCATATG	*csuC*
csuC R	GGATCGGCAACTCATCTACAATC	
csuE F	TCAGACCGGAGAAAAACTTAACG	*csuE*
csuE R	GCCGGAAGCCGTATGTAGAA	
pilT F	TTATTTTAGTCGGTGAGATGCGTG	*pilT*
pilT R	TCAATCACACGGTCAATGGTTT	
A1S_0113 F	GCCCTTTATTGGCAGATTTGAG	*A1S_0113*
A1S_0113 R	AGAGTCGCTTGCCAAAATCAC	
A1S_0115 F	CCTGTGACCTGTTGTCCCATT	*A1S_0115*
A1S_0115 R	TTCCTCGGTGGTTGGTGTTT	
A1S_0116 F	TTGATGCGGTGATTACTACCTGTTT	*A1S_0116*
A1S_0116 R	TGGTGAGCCGACTGATTTTG	

Supplementary Materials and Methods

Construction of the ΔbfmR mutant strain in A. baumannii ATCC 17978

The *A1S_0748* (*bfmR*) gene of *A. baumannii* ATCC 17978 was deleted using the markerless gene deletion method (Supplementary Figure S1A) [4]. The genomic DNAs purified from *A. baumannii* ATCC 17978 and pOH4 plasmid vector (Supplementary Table S1) were used for amplification of *A1S_0748* and kanamycin resistance cassette as PCR templates, respectively. Primers A1S_0748_SpeI UF, A1S_0748_ UR, A1S_0748_ DF, and A1S_0748_ DR (Supplementary Table S2) were designed using *A. baumannii* ATCC 17978 as a template. NptI_A1S_0748 F and NptI_ApaI R were designed using pOH4 as the template. Each gene fragment of *A1S_0748* upstream, *A1S_0748* downstream, and *npt*I were amplified using KOD FX NEO PCR enzyme (TOYOBO, Japan) using the respective templates and primers. The fragments were combined using overlap extension PCR. The combined DNA fragment was ligated into *Apa*I and *Spe*I-digested pDM4 vector using T4 DNA ligase (TOYOBO, Japan) (Supplementary Table S1). The pDM4 plasmids containing the combined DNA fragment were transformed into *E. coli* SY327 using the heat shock method. Transformants were selected on LB agar containing kanamycin and chloramphenicol. The plasmids were harvested using a plasmid mini-prep kit (GeneAll, Korea), and their sequences were analyzed. The plasmids were then transformed into *A. baumannii* ATCC 17978 cells by electroporation. Mutant bacteria that had undergone homologous recombination were selected on LB agar containing kanamycin as the first selection, followed by screening on LB agar containing 10% sucrose (Duksan, Korea). Deletion of *bfmR* in *A. baumannii* ATCC 17978 was confirmed by PCR analysis using A1S_0748_SpeI UF and A1S_0748_ DR primers (Supplementary Figure S1C). The Δ*bfmR* mutant strain was named HJ0748D (Supplementary Table S1).

Complementation of bfmR in the HJ0748D strain

The *bfmR* coding region with its native promoter and T1 terminator was inserted downstream of the *glmS* gene in the chromosome of the HJ0748 strain using overlap extension PCR (Supplementary Figure S1B). A DNA fragment, in which the *bfmR* coding region with its native promoter, T1 terminator, and upstream and downstream regions of the insertion site were combined by overlap extension PCR using specific primers. The primers glmS_up_ApaI F, glmS_up_A1S_0748 R, A1S_0748_glms_up F, and A1S_0748_T1 R were designed using *A. baumannii* ATCC 17978 as templates (Supplementary Table S2). T1_A1S_0748 F and npt I_Spe I R were designed using pOH4 as the template. The combined DNA fragments were ligated into *Spe*I- and *Apa*I-digested pDM4 (Supplementary Table S1). This plasmid was transformed into *E. coli* SY327 using the heat shock method, and the transformants were selected on LB agar containing kanamycin and chloramphenicol. The plasmids were purified using a plasmid mini-prep kit (GeneAll) and the sequences were analyzed. The plasmids were integrated into the chromosomes of the HJ0748D mutant strain by transformation and homologous recombination. Insertion of the *bfmR* coding region with its native promoter and T1 terminator was confirmed by PCR analysis using glmS_up_seq_F and glmS_down_seq_R primers (Supplementary Figure S1C). The *bfmR*-complemented strain was named HJ0748C (Supplementary Table S1).

Twitching motility assay

Twitching motility assays were performed on fresh-agarose plates (10 g tryptone/L, 5 g yeast extract, 5 g NaCl/L, 10 g agarose/L) prepared for each experiment. *A. baumannii* strains were inoculated by stabbing at the agarose and plate interface. The plates were incubated for 24 h at 37°C. Agarose was carefully removed from the plates and the plates were washed with phosphate-buffered saline (PBS). Bacteria adhered to the plates were stained with 0.1% crystal violet (wt/vol) in water for 5 min. The plates were washed with PBS and air-dried. Twitching motility area was measured using ImageJ program. The assays were performed in three independent experiments.

Bioassay for the detection of autoinducers

Agrobacterium minimal medium was prepared by mixing buffer A, buffer B, and distilled water. The composition of each buffer was as follows: buffer A, the PO_4 buffer (20 x), consisted of 60 g/L K_2HPO_4 and 20 g/L NaH_2PO_4; buffer B, the salt buffer (20 x), consisted of 20 g/L NH_4Cl, 6 g/L $MgSO_4.7H_2O$, 3 g/L KCl, 0.2 g/L $CaCl_2$, and 50 mg/L $FeSO_4.7H_2O$. The agar plate, used for the detection of autoinducers, was prepared by laying a bottom agar layer and then a top agar on plates. The bottom agar layer consisted of a mixture of buffer A (1 x), buffer B (1 x), 2% glycerol (*v*/*v*), carbenicillin (100 µg/mL), X-gal (Biopure, Daejeon, Korea) (100 µg/mL), and 1.5% Bacto agar (BD Difco, Franklin Lakes, NJ, USA) (*w*/*v*) dissolved in distilled water. The top agar layer consisted of a mixture of buffer A (1 x), buffer B (1 x), 2% glycerol (*v*/*v*), X-gal (100 µg/mL), and 1.2% Bacto agar (*w*/*v*) dissolved in distilled water. After pouring (16 mL) and solidification of the bottom agar layer in the plate, the top agar, containing *A. tumefaciens* NT1 (pDCI41E33) grown for 48 h, was added to the solidified agar. *A. baumannii* strains were grown overnight in LB at 37°C and then diluted, using fresh LB broth, to an OD_{600} of 1.0. Ten microliters of the diluted culture were loaded onto a plate overlaid with *A. tumefaciens* NT1 (pDCI41E33). The plates were then incubated for 22 h at 30°C. Autoinducer production was determined by measuring the colored zone surrounding the bacteria.

References

19 Kim, S.Y.; Kim, M.H.; Kim, S.I.; Son, J.H.; Kim, S.; Lee, Y.C.; Shin, M.; Oh, M.H.; Lee, J.C. The sensor kinase BfmS controls production of outer membrane vesicles in *Acinetobacter baumannii*. *BMC Microbiol.* **2019,** *19,* 301.

22 Oh, M.H.; Han, K. AbaR is a LuxR type regulator essential for motility and the formation of biofilm and pellicle in *Acinetobacter baumannii*. *Genes Genomics.* **2020,** *42,* 1339-1346.

3. Kwon, H.I.; Kim, S.; Oh, M.H.; Na, S.H.; Kim, Y.J.; Jeon, Y.H.; Lee, J.C. Outer membrane protein A contributes to antimicrobial resistance of *Acinetobacter baumannii* through the OmpA-like domain. *J. Antimicrob. Chemother.* **2017,** *72,* 3012-3015.

39 Oh, M.H.; Lee, J.C.; Kim, J.; Choi, C.H.; Han, K. Simple method for markerless gene deletion in multidrug-resistant *Acinetobacter baumannii*. *Appl. Environ. Microbiol.* **2015,** *81,* 3357-3368.
